# A novel mouse model for investigating α-synuclein aggregates in oligodendrocytes: implications for the glial cytoplasmic inclusions in multiple system atrophy

**DOI:** 10.1186/s13041-024-01104-7

**Published:** 2024-05-24

**Authors:** Tomoyuki Ishimoto, Miki Oono, Seiji Kaji, Takashi Ayaki, Katsuya Nishida, Itaru Funakawa, Takakuni Maki, Shu-ichi Matsuzawa, Ryosuke Takahashi, Hodaka Yamakado

**Affiliations:** 1 Department of Neurology, Graduate School of Medicine, Kyoto University, 54 Shogoin-Kawahara-Cho, Sakyo-Ku, Kyoto, 606-8507 Japan; 2 Department of Neurology, National Hospital Organization Hyogo-Chuo National Hospital, 1314 Ohara, Sanda, 669-1592 Japan

**Keywords:** Multiple system atrophy, MSA, α-synuclein, Glial cytoplasmic inclusions, GCI, Mouse model, Cellular tropism, Propagation, Prion hypothesis

## Abstract

The aggregated alpha-synuclein (αsyn) in oligodendrocytes (OLGs) is one of the pathological hallmarks in multiple system atrophy (MSA). We have previously reported that αsyn accumulates not only in neurons but also in OLGs long after the administration of αsyn preformed fibrils (PFFs) in mice. However, detailed spatial and temporal analysis of oligodendroglial αsyn aggregates was technically difficult due to the background neuronal αsyn aggregates. The aim of this study is to create a novel mouse that easily enables sensitive and specific detection of αsyn aggregates in OLGs and the comparable analysis of the cellular tropism of αsyn aggregates in MSA brains. To this end, we generated transgenic (Tg) mice expressing human αsyn-green fluorescent protein (GFP) fusion proteins in OLGs under the control of the 2’, 3’-cyclic nucleotide 3’-phosphodiesterase (CNP) promoter (CNP-SNCAGFP Tg mice). Injection of αsyn PFFs in these mice induced distinct GFP-positive aggregates in the processes of OLGs as early as one month post-inoculation (mpi), and their number and size increased in a centripetal manner. Moreover, MSA-brain homogenates (BH) induced significantly more oligodendroglial αsyn aggregates than neuronal αsyn aggregates compared to DLB-BH in CNP-SNCAGFP Tg mice, suggestive of their potential tropism of αsyn seeds for OLGs. In conclusion, CNP-SNCAGFP Tg mice are useful for studying the development and tropism of αsyn aggregates in OLGs and could contribute to the development of therapeutics targeting αsyn aggregates in OLGs.

**Supplementary Information:**

The online version contains supplementary material available at 10.1186/s13041-024-01104-7.

## Introduction

Multiple system atrophy (MSA) is a neurodegenerative disease that is clinically characterized by various symptoms, including parkinsonism, cerebellar ataxia, and autonomic dysfunction [1, 2]. The main pathological findings of MSA are the presence of alpha-synuclein (αsyn) aggregates in oligodendrocytes (OLGs) called glial cytoplasmic inclusions (GCIs) and neuronal loss, but neuronal cytoplasmic inclusions (NCIs) are also observed [3, 4]. αsyn aggregates in MSA are mainly formed in OLGs, but almost exclusively formed in neurons in Parkinson’s disease (PD) and dementia with Lewy bodies (DLB) [5–13]. However, the mechanism by which αsyn preferentially accumulates in OLGs in MSA remains unclear, even though the expression level of αsyn in OLGs is much lower than that in neurons [14–17].

There is growing evidence suggesting that αsyn has a prion-like property. The injection of αsyn preformed fibrils (PFFs) into mouse brains resulted in the propagation of αsyn in neurons [18–23], and injection of MSA brain lysates into mouse brains also caused the propagation of αsyn exclusively in neurons, but not in OLGs [24, 25]. This result further deepened the mystery of how αsyn accumulates in OLGs in MSA.

We previously reported that αsyn accumulates not only in neurons but also in OLGs following long-term incubation (at least 7 months) in mice inoculated with αsyn PFFs [26]. However, the process of early αsyn aggregate formation in OLGs was difficult to be evaluated with the phosphorylated αsyn antibody due to the overwhelming background signal generated by αsyn aggregates in neurons.

The aim of this study was to create a mouse model that enables the sensitive and specific detection of αsyn aggregates in OLGs and the analysis of their preferential accumulation in OLGs. To the best of our knowledge, this is the first study to precisely analyze the spatial and temporal development of oligodendroglial αsyn aggregates in vivo.

## Results

### Oligodendrocyte-specific αsynGFP expression in CNP-SNCAGFP Tg mice

**Fig. 1:**
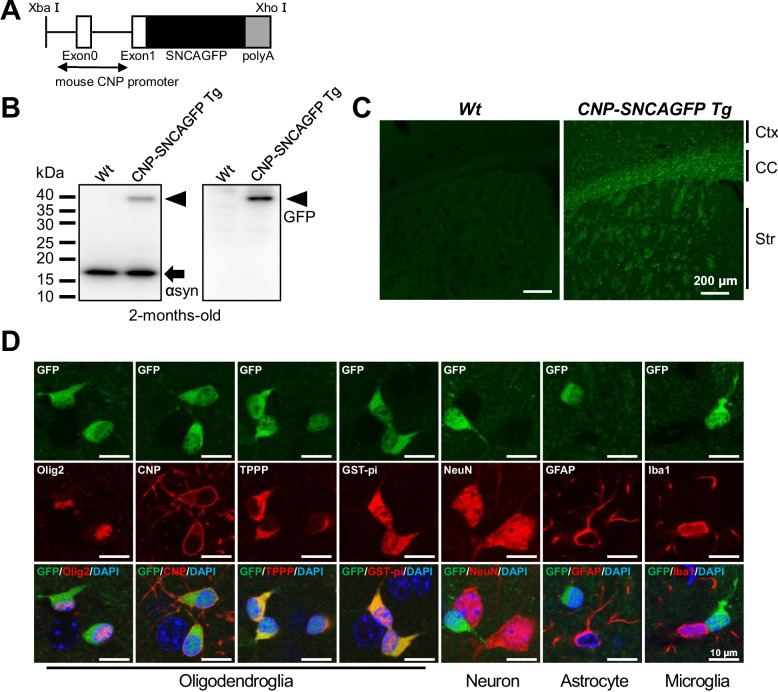
The expression of human αsynGFP in CNP-SNCAGFP transgenic (Tg) mice. **A** The transgenic construct. **B** Western blot images of whole-brain SDS-soluble fraction with anti-αsyn and GFP antibodies in both genotypes (2 months old). The expression of endogenous αsyn (arrow) and transgenic human αsynGFP fusion protein (arrowhead) is shown. **C** Fluorescence microscopy showed the αsynGFP expression in in both genotypes (2 months old). Scale bar = 200 µm. **D** Immunofluorescent staining of various cellular makers with αsynGFP in the striatum of Tg mice (2 months old). The merged images include DAPI (blue). GFP signals were observed exclusively in OLGs. Note that anti-CNP antibodies strongly labeled the plasma membrane in OLGs. Scale bar = 10 µm. Wt, wild-type; αsyn, alpha-synuclein; Ctx, cortex; CC, corpus callosum; Str, striatum

We created transgenic (Tg) mice expressing human αsyn-GFP fusion proteins in OLGs by the murine CNP promoter (CNP-SNCAGFP Tg mice) (Fig. 1A). The CNP promoter was employed because αsyn has been reported to accumulate in mature OLGs as well as oligodendrocyte precursor cells (OPCs) in MSA, and CNP is known to be expressed in OPCs [27, 28]. OPCs were also reported to have a greater ability to incorporate αsyn aggregates compared to mature OLGs and contribute to the development of GCIs [29]. In addition to the endogenous αsyn expression, the human αsyn-GFP fusion protein was expressed in CNP-SNCAGFP Tg mice (Fig. 1B). A histological study showed that αsynGFP was robustly expressed in the corpus callosum, moderately expressed in the striatum and white matter of the cerebral cortex, and mildly expressed in the gray matter of the cerebral cortex (Fig. 1C). To examine the cells expressing αsynGFP in CNP-SNCAGFP Tg mice, immunofluorescent staining with antibodies against markers of OLGs, neurons, astrocytes, and microglia was performed. GFP co-localized with Olig2, CNP, TPPP, and GSTpi, but not with NeuN, GFAP, or Iba1, confirming that αsynGFP is only expressed in OLGs (Fig. 1D). αsynGFP was observed mainly in the cell bodies and also in the process of OLGs in CNP-SNCAGFP Tg mice (Fig. 1D). Note that the subcellular distribution of αsynGFP in CNP-SNCAGFP Tg mice was the same to that of human αsyn in CNP-SNCA Tg mice (Additional file 1: Fig. S1A). Furthermore, the phosphorylation or aggregation status of αsynGFP did not change with aging (Additional file 1: Fig. S1B, Additional file 2: Fig. S2C left panel), and these mice did not show apparent motor impairment even at 12 months of age (Additional file 1: Fig. S1C).

### Highly sensitive detection of αsyn aggregates in OLGs in CNP-SNCAGFP Tg mice

We have previously shown that in mice inoculated with αsyn PFFs, the accumulation of αsyn in OLGs was observed long after inoculation [26]. In the present study, we tested whether the temporal development of αsyn aggregates in OLGs could be monitored by the GFP signals in Tg mice according to the same paradigm.

**Fig. 2:**
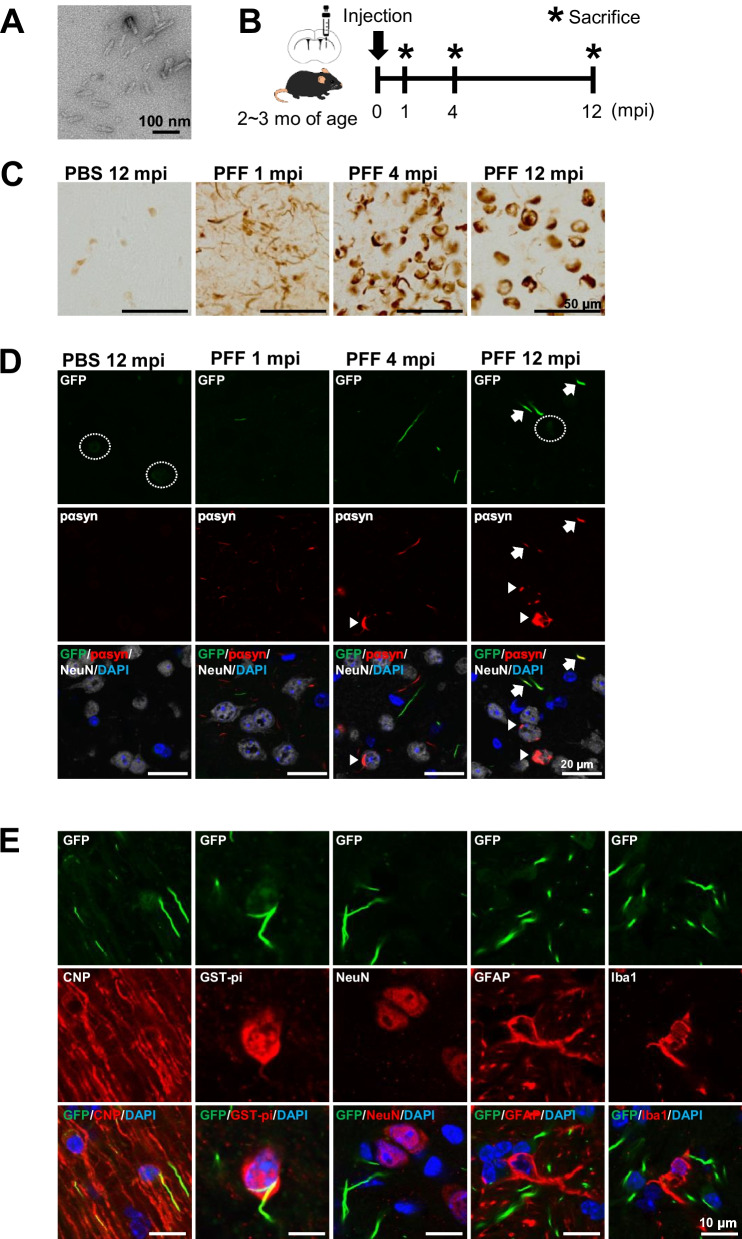
The development of pαsyn and αsynGFP aggregates in CNP-SNCAGFP Tg mice inoculated with αsyn PFFs. **A** Image of sonicated αsyn PFFs used for injection in electron microscopy. **B** The schedule of αsyn PFF injection and histological evaluation. Tg mice (age: 2–3 months) were inoculated with αsyn PFFs or PBS, sacrificed at 1, 4, and 12 mpi, and histological examinations were performed (*n* = 6). **C** Immunohistochemical staining for pαsyn at the ipsilateral dorsal striatum of Tg mice inoculated with αsyn PFFs or PBS. In Tg mice, numerous pαsyn-positive neurites were observed as early as 1 mpi, and these thread-like structures grew into round-shaped cytoplasmic inclusions. Only a few pαsyn-positive cells were observed in Tg mice inoculated with PBS. Scale bar = 50 µm. **D** Fluorescence micrographs of GFP and immunostaining with anti-pαsyn (red) and anti-NeuN (gray) antibodies in the striatum of Tg mice. The merged images include DAPI (blue). Unaggregated αsynGFP is denoted by dotted circles. Most of the pαsyn-positive signals are of neuronal origin (arrowheads), and rarely merged with αsynGFP aggregates (GFP dots) in OLGs (arrows). Scale bar = 20 µm. **E** Immunofluorescence micrographs of various cellular makers with GFP in Tg mice at 6 mpi of αsyn PFFs. αsynGFP aggregates were observed specifically in the OLGs. The merged images include DAPI (blue). Scale bar = 10 µm. mpi, month(s) post-inoculation; PFFs, preformed fibrils; pαsyn, phosphorylated α-synuclein

Electron microscopy confirmed that αsyn PFFs were fragmented to about 20–50 nm by sonication (Fig. 2A). αsyn PFFs or PBS were inoculated in the left dorsal striatum of CNP-SNCAGFP Tg mice and histological analyses were performed at 1, 4, and 12 months after inoculation (Fig. 2B). Numerous phosphorylated αsyn (pαsyn)-positive aggregates were observed as early as 1-month post-inoculation (mpi), and these thread-like structures developed into round-shaped cytoplasmic inclusions (Fig. 2C). Although most of the pαsyn-positive aggregates were colocalized with neuronal markers and of neuronal origin, pαsyn and GFP positive αsyn aggregates in OLGs were also identified. However, weak and rare pαsyn-positive signals in OLGs were buried in strong background signals from neuronal aggregates in most of the brain regions, except for the corpus callosum where OLGs outnumber neurons (Fig. 2D, Additional file 3: Fig. S3).

To confirm that αsynGFP aggregates, which we call GFP dots, were of oligodendroglial origin, immunofluorescent staining with antibodies against various cell-specific markers was performed. GFP dots co-localized with CNP and GSTpi, but not NeuN, GFAP, or Iba1, confirming that GFP dots were formed specifically in OLGs, and not in other cell types by the leakage or transfer of αsynGFP from OLGs (Fig. 2E). GFP dots were immunopositive for ubiquitin (Ub), p62/sequestrosome-1 (p62), aggregated form of αsyn, and were PK resistant, suggesting that these are pathological aggregates (Additional file 2: Fig. S2).

### Spatial and temporal distribution of αsyn aggregates in neurons and OLGs in CNP-SNCAGFP Tg mice inoculated with αsyn PFFs

**Fig. 3:**
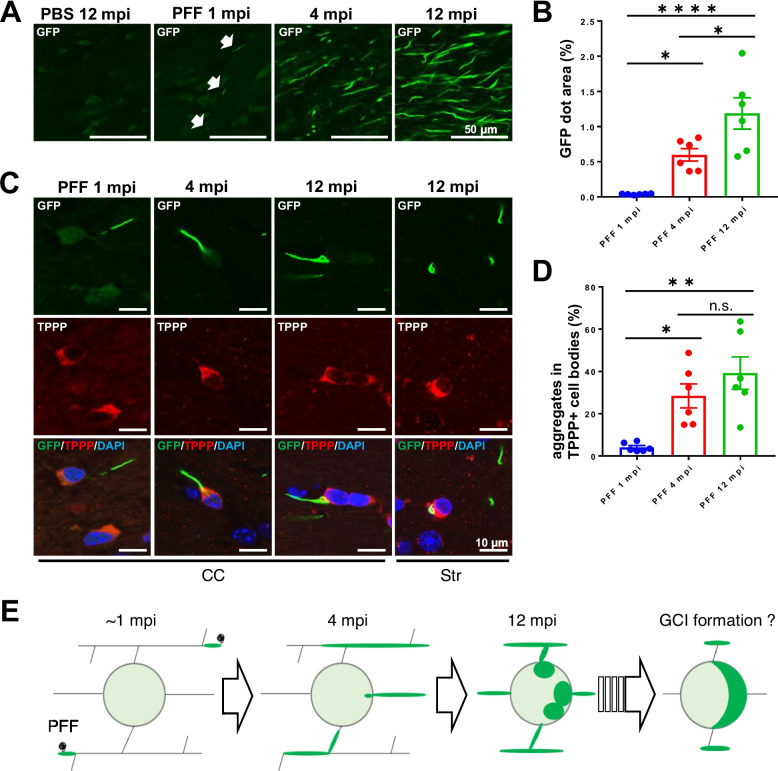
αsynGFP aggregates in OLGs developed in a centripetal manner in CNP-SNCAGFP Tg mice inoculated with αsyn PFFs. **A** Fluorescence micrographs of the corpus callosum of Tg mice inoculated with αsyn PFFs. Small αsynGFP aggregates (GFP dots) were observed as early as 1 mpi (arrows), and their size and signal intensity increased over time. Scale bar = 50 µm. **B** Quantification of the GFP dot area in (**A**) at 1, 4, and 12 mpi (*n* = 6). **C** Fluorescence micrographs of GFP and immunostaining with anti-TPPP antibody in the corpus callosum of Tg mice at 1, 4, and 12 mpi. The merged images include DAPI (blue). GFP dots were initially observed in the OLG process and developed toward the cell body. Scale bar = 10 µm. **D** Quantification of the number of aggregates in TPPP-positive cell bodies in (**C**) at 1, 4, and 12 mpi (*n* = 6 at each time point). **E** The development of GFP dots is illustrated. Tukey’s multiple comparisons test was performed in (**B**) and (**D**); *p < 0.05, **p < 0.01, ****p < 0.0001, n.s., not significant. Data indicate the mean ± SEM. mpi, months post-inoculation; CC, corpus callosum; Str, striatum

The GFP dots in mice inoculated with αsyn PFFs represented the αsyn aggregates in OLGs. They were mainly observed in the corpus callosum, followed by the striatum, and white matter of the cerebral cortex (Additional file 3: Fig. S3A). They first appeared as early as 1 mpi of αsyn PFFs, and their signal intensity increased over time until 12 mpi (Figs. 2D, and 3A, Additional file 3: Fig. S3A). In contrast, the signal intensity of pαsyn reached a plateau at 4 mpi and remained stable until 12 mpi. These pαsyn-positive aggregates rarely colocalized with the GFP dots (Additional file 3: Fig. S3B), and were sparse in OLG-rich regions, suggesting their neuronal origin (Additional file 3: Fig. S3A). Although majority of GFP-positive structures within OLGs also become pαsyn -positive with increased sensitivity (Fig. S3C), this makes the analysis more difficult due to the strong pαsyn signal of neuronal origin, especially in the striatum and cerebral cortex where neurons outnumber OLGs. These results suggest that, compared to αsyn phosphorylation, GFP dot signals exhibit much higher sensitivity in detecting αsyn aggregates in OLGs, especially in the early stage and regions abundant in neurons.

### Development of αsynGFP aggregates in a centripetal manner in CNP-SNCAGFP Tg mice inoculated with αsyn PFFs

αsynGFP aggregates (GFP dots) were first observed at 1 mpi, and their number, size, and signal intensity increased over time in CNP-SNCAGFP Tg mice inoculated with αsyn PFFs (Fig. 3A, B). They were initially formed in the process far away from the TPPP-positive cell bodies in OLGs at 1 mpi (Fig. 3C). However, they developed in a centripetal manner and some of them became visible in TPPP-positive cell bodies in the striatum at 12 mpi (Fig. 3C, D). Figure 3E provides an illustrated summary of the development of GFP dots in these mice.

### Thread-like αsyn aggregates in the processes of OLGs in the brains of patients with MSA

**Fig. 4:**
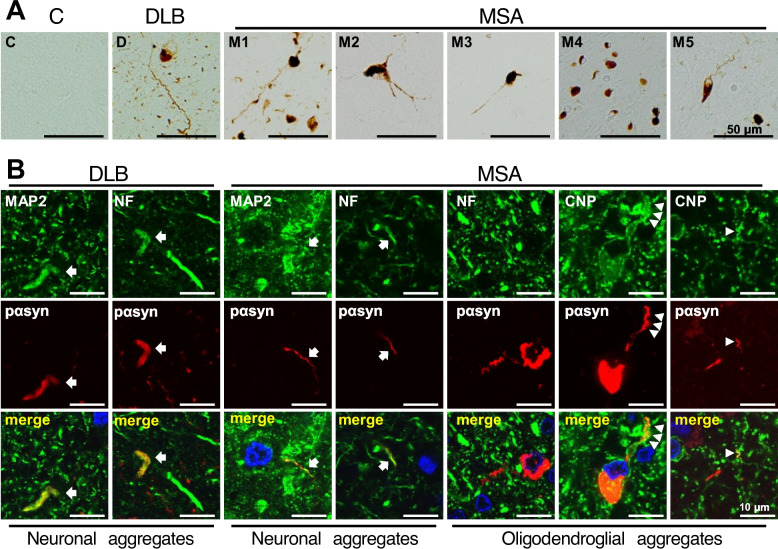
Thread-like αsyn aggregates in the process of OLGs in MSA brains. **A** Immunohistochemical staining of human autopsy brains with anti-pαsyn antibody. Some of the pαsyn-positive aggregates in MSA brains showed thread-like structures continuous with the cytoplasmic inclusions. The following regions were analyzed: the frontal lobes (C, D, M1-3), the putamen (M4), and the cerebellum (M5). Scale bar = 50 µm. **B** Double immunofluorescent staining of thread-like aggregates with anti-pαsyn antibody and various cellular markers in DLB and MSA brains. The merged images include DAPI (blue). Lewy neurites in DLB brains and some of the thread-like pαsyn-positive aggregates in MSA brains co-localized with MAP2 and NF (arrows). Other pαsyn-positive aggregates in MSA brains were not co-localized with neuronal makers but were co-localized with CNP and were continuous with GCIs (arrowheads). Note that anti-CNP antibodies strongly labeled the plasma membrane in OLGs. Scale bar = 10 µm. C, control; D, DLB (dementia with Lewy bodies); M, MSA. NF, neurofilament; pαsyn, phosphorylated α-synuclein

The thread-like pαsyn-positive structures were observed in the postmortem brains of patients with MSA (Fig. 4A), some of which co-localized with neuronal markers and were of neuronal origin. However, others did not co-localize with neuronal markers but co-localized with CNP, and were therefore of oligodendroglial origin (Fig. 4B). In contrast, in brains of patients with DLB, pαsyn-positive aggregates co-localized with neuronal markers MAP2 and neurofilament, but not with CNP (Fig. 4B and Additional file 4: Fig. S4). These results suggest that some of the GCIs may developed from small αsyn aggregates in the processes of OLGs in MSA, as shown in CNP-SNCAGFP Tg mice inoculated with αsyn PFFs.

### MSA brain homogenates (BH) induced more αsyn aggregates in OLGs than DLB BH in CNP-SNCAGFP Tg mice

**Fig. 5:**
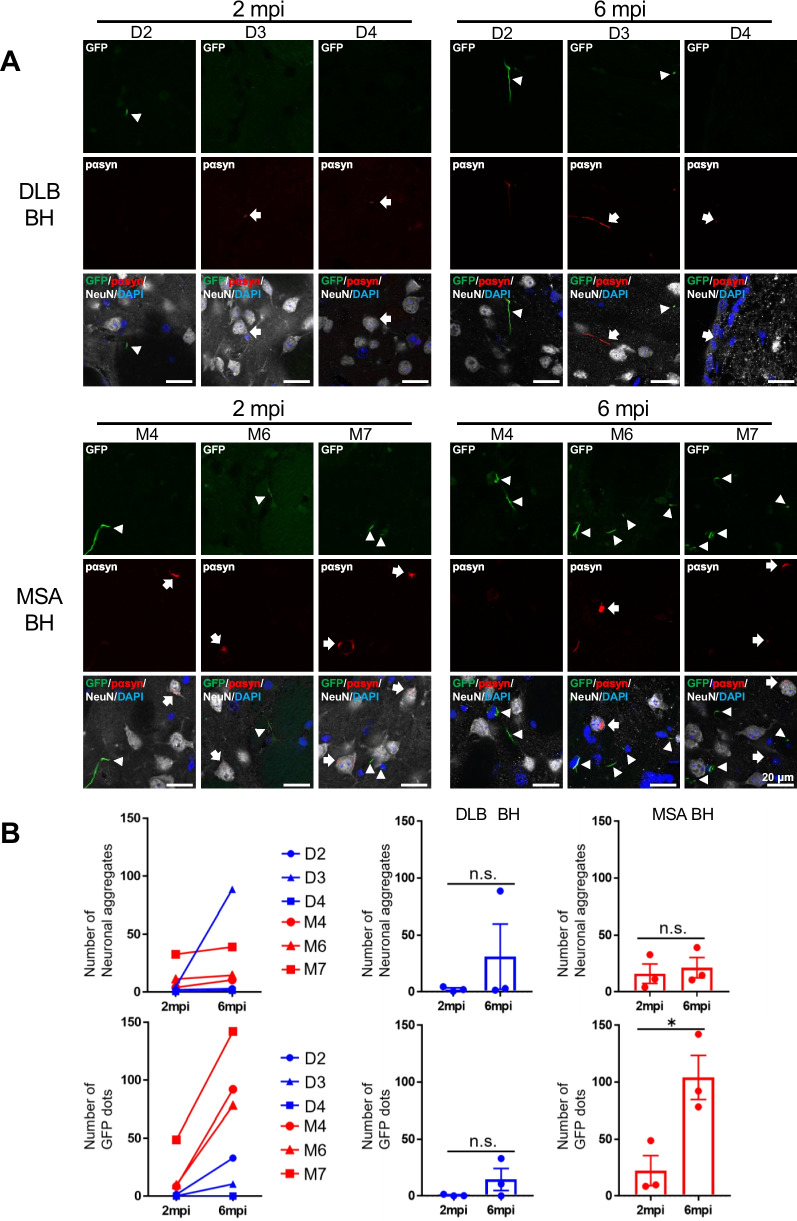
Inoculation of brain homogenates from MSA samples induced distinct αsyn aggregates in OLGs in CNP-SNCAGFP Tg mice. **A** Fluorescence micrographs of GFP and immunostaining with anti-pαsyn (red) and anti-NeuN (gray) antibodies in the striatum of Tg mice treated with BH from DLB or MSA samples at 2 mpi and 6 mpi. The merged images include DAPI (blue). GFP dots (arrowheads) represent aggregates in OLGs. Aggregates that were immunopositive for pαsyn and not co-localized with GFP were neuronal (arrows). **B** Comparison of the number of αsyn aggregates in neurons and OLGs in the striatum (bregma + 0 mm) of Tg mice treated with BH from DLB or MSA. A single data point represents the average number of αsyn aggregates of four mice at 2 mpi and two mice at 6 mpi, respectively. Neither the neuronal aggregate nor GFP dot exhibited a significant increase over time in the DLB BH-treated group. In contrast, the MSA BH-treated group displayed a notable increase only in GFP dots at 6 mpi. Scale bar = 20 µm. Student’s t-test was performed in (**B**); *p < 0.05, n.s., not significant. Data indicate the mean ± SEM. BH, brain homogenates; D, DLB (dementia with Lewy bodies); M, MSA; mpi, months post-inoculation; pαsyn, phosphorylated α-synuclein

To investigate the tropism of αsyn aggregates for OLGs in MSA, CNP-SNCAGFP Tg mice were inoculated with MSA and DLB BH and analyzed at 2 and 6 mpi. αsyn aggregates in OLGs were identified by GFP dots and those in neurons were identified by pαsyn-positivity and GFP-negativity (Fig. 5A). When the numbers of neuronal and oligodendroglial αsyn aggregates were compared between 2 and 6 mpi, there was no significant increase in the number of neuronal or oligodendroglial αsyn aggregates in the DLB BH-treated group. In contrast, in the MSA BH-treated group, there was no significant increase in the number of neuronal αsyn aggregates, but there was a significant increase in the number of oligodendroglial αsyn aggregates, suggestive of the tropism for OLGs (Fig. 5B).

## Discussion

How GCIs are formed and develop in MSA has remained a great mystery. Cases with preclinical MSA or incidental GCIs might contribute to answering this question [30, 31], but the number of such cases is limited and thus an animal model is an attractive option.

There are already excellent mouse models showing αsyn pathology and associated motor symptoms. The model that expresses αsyn under the PLP promoter show αsyn pathology in OLGs and age-dependent motor symptoms as well as autonomic dysfunction [32–35]. Mouse models expressing αsyn under the myelin basic protein (MBP) promoter are also useful models showing αsyn pathology, motor symptoms, and neuroinflammation [36, 37]. M2 mice, which express human αsyn under the CNP promoter, show phosphorylation of αsyn in OLGs and age-dependent motor symptoms [38]. Furthermore, αsyn seed administration to KOM2, a crossbreed between endogenous αsyn knockout mice and M2 mice, induces αsyn aggregate formation in the OLGs [39]. However, animal model specifically designed to explore the process and tropism of aggregate formation within OLGs is still lacking.

In this study, we succeeded in visualizing the aggregation process of endogenous αsyn in OLGs from an early stage by employing newly generated CNP-SNCAGFP Tg mice inoculated with αsyn PFFs. The sensitive, specific and early detection of αsyn aggregates by GFP dots overcame the technical difficulties in identifying αsyn aggregates, most of which are phosphorylated, in OLGs in the presence of numerous neuronal p-αsyn aggregates. Indeed, we previously reported that αsyn aggregates in OLGs were observed at 7 mpi of αsyn PFFs in wild-type mice, as assessed by pαsyn antibodies [26], whereas in the present study they could be detected as early as 1 mpi. In these mice, endogenous αsyn aggregates were initially observed at the process and they developed toward the cell body in OLGs. This centripetal development of aggregates is in part consistent with findings in postmortem brains of MSA patients, and is not likely an effect of the fused GFP protein because the distribution of human αsynGFP did not differ from that of human αsyn in CNP-SNCA Tg mice.

The origin of αsyn aggregates in MSA has been considered to be OLGs, because in most cases GCIs appeared earlier and were more abundant than neuronal aggregates. Contrary to this notion that MSA is a primary oligodendrogliopathy, it has recently been reported that αsyn aggregates in neurons are more widespread than previously thought [3, 5, 40, 41], and that in some cases of MSA, they are predominantly found in neurons of specific brain regions [5]. αsyn oligomers visualized by an αsyn proximity ligation assay (PLA) are also reported to be observed in cortical neurons in the early stages of MSA [42]. Taken together with the fact that the expression level of αsyn in OLGs is much lower than that in neurons, these studies strongly suggest the neuronal origin of αsyn aggregates in GCIs [43]. There are also two possibilities for the origin of αsyn seeds in OLGs in our mouse model. One is that αsyn aggregates in OLGs directly seeded by inoculated αsyn PFFs. This is supported by previous studies that upon treatment of αsyn PFFs, αsyn aggregates formed within primary OPCs [29] and in OLGs of mice that express human αsyn exclusively in OLGs in a murine αsyn null background [39]. These reports suggest that αsyn aggregates can be taken up by and develop in OPCs/OLGs without neuronal αsyn. The other possibility is that they propagated from αsyn aggregates in neurons that were seeded by injected αsyn PFFs. Although αsyn aggregates were observed in OLGs at as early as 1 mpi of αsyn PFFs, numerous αsyn aggregates were already observed in neurons by this time. Therefore, it is difficult to determine whether the αsyn aggregates in OLGs in this model are of neuronal origin or not. Another limitation is that the oligodendroglial aggregates observed in this model were less stained by antibodies for pαsyn, possibly due to the fused GFP protein at the C-terminus of transgenic human αsyn [44]. It is well known that the aggregation process of GFP fusion proteins is often different from that of naked proteins. In the case of αsyn, it has been reported that αsyn-GFP aggregates exhibit a fibril structure similar to that of naked protein aggregates by electron microscopic analysis [44], but the aggregation propensity of αsyn-GFP is somewhat reduced compared to naked protein due to the presence of GFP at the C-terminus [44]. Therefore, physicochemical properties of αsyn-GFP may be similar but not entirely identical to those of the naked αsyn protein. Despite these limitations, this model may still be valuable for the sensitive and specific monitoring of the development of αsyn aggregates in OLGs.

The properties of αsyn aggregates have been shown to differ pathologically between PD, DLB, and MSA [45], and αsyn aggregates amplified from patient blood samples also differ biochemically between PD, DLB, and MSA [46]. Recent studies showed that αsyn seeds generated in the different cellular milieu cause different pathologies depending on their strains [39, 47, 48], and there may be αsyn seeds that preferentially affect OLGs. To address this issue, we inoculated BH from MSA and DLB samples in CNP-SNCAGFP Tg mice. In contrast to many previous studies showing that MSA BH induced the αsyn aggregates exclusively in neurons in wild-type and αsyn Tg rodents [24, 49, 50], not only MSA BH but also DLB BH induced αsyn aggregates in OLGs. This can be explained by the overexpressed human αsyn in OLGs and is consistent with the recent report showing that BH induced αsyn aggregates in OLGs of P1 artificial chromosome (PAC) transgenic mice which overexpress human αsyn in OLGs by the endogenous αsyn promoter [51]. In our study, MSA BH induced more aggregates than DLB BH in neurons and OLGs at 2 mpi. This may be due to the higher seeding activities of MSA BH, but the greater increase in the number of aggregates in OLGs compared to neurons at 6 mpi can be explained by the tropism of αsyn seeds in MSA for OLGs. These studies demonstrate that CNP-SNCAGFP Tg mice will be useful for investigating the cell-type preference of αsyn seeds by sensitive detection in OLGs.

In conclusion, we generated a novel mouse model that enables the sensitive and specific detection of αsyn aggregates in OLGs from an early stage. CNP-SNCAGFP Tg mice are expected to help investigate the formation of GCI and thus be useful as a preclinical model for the development of therapeutic agents, such as inhibitors of αsyn aggregation in OLGs.

## Methods

### Animals

The mice used in this study were handled according to national guidelines. All procedures performed in this study were approved by the Institutional Animal Care and Use Committee of the Laboratory Animal Research Institute, Kyoto University Graduate School of Medicine (Med Kyo 18,215).

The transgenic construct of the CNP-SNCAGFP Tg mouse and CNP-SNCA Tg mouse were generated as follows. The coding sequence of human αsynGFP or αsyn was cloned into the pBSK plasmid harboring the murine 2',3'-cyclic nucleotide 3'-phosphodiesterase (CNP) promoter [52], which was a generous gift from Dr. Vittorio Gallo (Children’s National Research Institute, DC). Next, the ligated plasmid was digested with XbaI and XhoI, purified and microinjected into C57BL6/J fertilized eggs. For genotyping, the following primer sets was used: Forward 5´- GGCTGGCTTTGAGGAGCC-3´, Reverse 5´-GGGCTCCTTCTTCATTCTT-3´. Homozygous Tg was used in brain homogenate experiments and real-time PCR was performed to identify homozygous Tg using the following primer sets: Forward 5´-AAGTTCATCTGCACCACCG-3´, Reverse 5´-TCCTTGAAGAAGATGGTGCG-3´.

### Human brain samples

Autopsied brains of patients with MSA, DLB, and non-neurodegenerative disease controls were used (listed in Additional file 5: Table S1). Frozen tissue samples were obtained from Kyoto University Hospital, National Hospital Organization Hyogo-Chuo National Hospital, and the University of California, San Diego. Frozen human brain tissues were homogenized in PBS to 10% (wt/vol) and centrifuged at 2,000 × g for 10 min at 4 ℃. The supernatant was used for inoculation. All procedures using human materials were performed in accordance with the ethical guidelines and approved by the Ethics Committee of Kyoto University Graduate School and Faculty of Medicine (R1038).

### Immunohistochemical analysis of mouse and human brain samples

Mice were anesthetized with sevoflurane and transcardially perfused with phosphate-buffered saline (PBS), followed by 4% (w/v) paraformaldehyde in PBS. The brains were then removed and immersed in 4% (w/v) paraformaldehyde in PBS overnight at 4°C, replaced with 30% sucrose (w/v) in PBS, embedded in O.C.T compound (SAKURA Finetek), and frozen at -80°C. The mice brains were sliced in 40-µm-thick in a cryostat and stored in 0.02% NaN_3_ (w/v) in PBS at 4°C. In addition, formalin-fixed, paraffin-embedded, 8-μm-thick brain sections were used for staining with antibodies for αsyn aggregates. For the immunohistochemical analysis of human brains, formalin-fixed, paraffin-embedded, 6-μm-thick sections were deparaffinized and heat-induced antigen retrieval was performed with Tris–EDTA buffer (pH 9.0).

Sections were immersed in 3% hydrogen peroxide in PBS for 30 min at room temperature (RT) to inactivate the endogenous peroxidase activity. After blocking with 10% goat serum and 0.02% Triton-X 100 in PBS for 1 h at RT, the sections were incubated overnight at 4°C with the primary antibody (listed in Additional file 5: Table S2). Then, the sections were incubated with peroxidase-labeled secondary antibodies (Histofine Simplestain Max PO, Nichirei Biosciences) and visualized by diaminobenzidine staining (Nacalai Tesque).

### Immunofluorescence staining

Formalin-fixed, paraffin-embedded sections were deparaffinized, and heat-induced antigen retrieval was performed with Tris–EDTA buffer (pH 9.0). This process was not performed on free-floating mouse brain sections. After blocking with 10% goat or donkey serum and 0.02% Triton-X 100 in PBS for 1 h at RT, sections were incubated overnight at 4°C with primary antibodies (listed in Additional file 5: Table S2) (when MJFR-14–6-4–2 antibody was used, 0.5 M NaCl was added to the buffer). These sections were incubated with goat- or donkey-derived secondary antibodies (Alexa Fluor 488, 594, and 647, 1:400, Thermo Fisher) for 2 h at RT. The sections were incubated with Vector TrueVIEW Autofluorescence Quenching Kit (Vector Laboratories) for 5 min to reduce autofluorescence, covered with Vibrance Antifade Mounting Medium with DAPI (Vector Laboratories), and observed with an FV1000 confocal microscope (OLYMPUS).

### Proteinase K digestion

Proteinase K (PK) digestion was used to determine solubility of the human αsyn observed in OLGs with minor modifications [53]. Striatal sections were mounted onto FRONTIER-coated slide glass (Matsunami glass) and dried overnight at RT. After washing with PBS, the sections were digested with 200 μg/ml PK in PBS for 30 min at RT. After several washes, the sections were immunostained as described above.

### Generation of recombinant mouse αsyn monomers and fibrils

Recombinant αsyn monomers were generated as previously described, with minor modifications [26, 54, 55]. Briefly, mouse αsyn was expressed in *Escherichia coli* BL21 (DE3) (BioDynamics Laboratory) and purified by ion exchange using Q Sepharose Fast Flow (GE Healthcare). Endotoxin from *Escherichia coli* was removed using the ToxinEraser™ Endotoxin Removal Kit (GenScript), and the endotoxin levels were confirmed to be below the detection sensitivity with LAL Endotoxin Assay Kit (Genscript). After dialysis against 5 mM Tris–HCl and the addition of 10 × PBS to make 1 × PBS solution, αsyn monomers (5 mg/ml) were incubated at 37°C with constant agitation at 1,000 rpm for 7 days. The solution was sonicated for 5 min with a Bioruptor (Sonicbio) before injection.

### Western blotting

Western blot analyses were conducted as described previously with minor modifications [56]. Briefly, mice brains perfused with cold PBS were homogenized in a tenfold volume of lysis buffer (150 mM NaCl, 1 mM ethylenediaminetetraacetic acid, 10 mM Tris–HCl, 1% (v/v) TritonX-100, 2% (v/v) SDS) or RIPA buffer (20 mM HEPES pH 7.4, 150 mM NaCl, 2 mM EDTA, 1% (v/v) Nonidet-P40, 0.5% (v/v) sodium deoxycholate,0.1% (v/v) SDS). The homogenates were centrifuged at 100,000 × *g* for 30 min at RT. The supernatant was boiled in sample buffer (1% (w/v) SDS, 12.5%(w/v) glycerol, 0.005% (w/v) bromophenol blue, 50 mM dithiothreitol, 25 mM Tris–HCl, pH 6.8), and 5 μg of protein was loaded and separated by SDS-PAGE 5–20% (w/v) gradient gels (Wako). Proteins were transferred to polyvinylidene difluoride membranes using Trans-Blot SD Semi-Dry Transfer Cell (Bio-Rad). The membranes were treated with 4% (w/v) paraformaldehyde in Tris-buffered saline (TBS) for 30 min at RT. After blocking for 1 h with 5% (w/v) skim milk in TBS with 0.1% (v/v) Tween 20 (TBST), each membrane was incubated with anti-αsyn (BD Bioscience, clone 42, 1:2,000), anti-GFP (Abcam, EPR14104, 1:10,000), and anti-pαsyn (Abcam, EP1536Y, 1:10,000) antibodies overnight at 4°C. These membranes were washed with TBST and incubated with horseradish peroxidase-conjugated secondary antibodies (NB7574 or NB7160, Novus Biologicals,1:10,000) for 1 h. Images were acquired using an Amersham Imager 600 (GE Healthcare).

### Transmission *electron* microscopy

Sonicated αsyn PFFs were dropped onto a 200-mesh carbon-coated copper grid (Nissin EM), and PFFs adsorbed on the grid were negatively stained with 1% (w/v) uranyl acetate solution. Electron micrographs were obtained using a transmission electron microscope (H-7650, HITACHI) at 80 kV.

### Intracerebral injection of αsyn PFFs and brain homogenate (BH)

CNP-SNCAGFP Tg mice (age: 2–3 months) were anesthetized with isoflurane and inoculated with 2 μl (10 μg) of αsyn PFFs (*N* = 20), 2 μl PBS (*N* = 3) or 2 μl BH (*N* = 18) into the left dorsal striatum (A/P, + 0.2 mm; M/L, + 2.0 mm; D/V, − 2.6 mm) using a 33-gauge Neuros syringe (Hamilton).

### Statistical analysis

All statistical analyses were performed using the GraphPad Prism software program (version 7.04). All statistical tests performed are described in Figure Legends. *P* values of < 0.05 were considered to indicate statistical significance.

### Supplementary Information

Additional file 1: Fig. S1. The αsynGFP in CNP-SNCAGFP Tg mice exhibits similar subcellular distribution to αsyn in CNP-SNCATg mice and does not aggregate with age. (A) Fluorescence micrographs of GFP and immunostaining with anti-human αsyn antibodies (red) and CNP (gray) of the striatum in CNP-SNCAGFP Tg mice and in CNP-SNCA Tg mice that express human αsyn in OLGs. Scale bar = 10 µm. (B) Western blot images of RIPA-soluble fraction with anti-αsyn and pαsyn antibodies in whole brains of Wt and Tg mice (2, 13, and 18 months old). The expression of endogenous mouse αsyn (arrow) and transgenic human αsynGFP fusion protein (arrowhead) are shown. (C) Latency to fall in the rotarod test at 12 months old. Student’s t-test was performed (not significant). Data indicate the mean ± SEM. Wt, wild-type; αsyn, alpha-synuclein; pαsyn, phosphorylated α-synuclein; PK, proteinase K; RT, room temperature.

Additional file 2: Fig. S2. GFP dot signals co-localized with markers of αsyn aggregates. (A) Fluorescence micrographs of GFP and immunostaining with anti-ubiquitin, p62, and aggregated form of αsyn antibodies (MJFR-14–6-4–2) in the striatum of Tg mice at 6 mpi of αsyn PFFs. The merged images include DAPI (blue). Aggregates that were immunopositive for p62 and MJFR-14–6-4–2 that did not co-localized with GFP were neuronal (arrowheads). (B) Immunohistochemical staining with anti-GFP and aggregated form of αsyn antibodies (5G4 and A17183A) in the striatum of Tg mice at 6 mpi of αsyn PFFs (paraffin-embedded sample). The merged images include DAPI (blue). Scale bar = 10 µm. (C) Immunohistochemical staining with human-specific αsyn antibody (MJFR1) in the striatum of 2, 12, and 20 months-old Tg mice (left panel) and in the striatum of Tg mice at 6 mpi of PBS or αsyn PFFs (right panel). The lower panel shows sections stained under the same conditions as the upper panel after PK digestion (200 µg/ml, 30 min, RT). Arrows represent αsyn aggregates in OLGs. Scale bar = 20 µm. Ub, ubiquitin; PK, proteinase K; RT, room temperature.

Additional file 3: Fig. S3. Sensitive detection of oligodendroglial aggregates by αsynGFP signals in CNP-SNCAGFP Tg mice treated with αsynPFFs. Fluorescence micrographs of GFP and immunostaining with pαsyn antibodies in Tg mice (low-power magnification). The signal intensity of GFP dots increased over time until 12 mpi, especially in the CC, whereas that of pαsyn reached a plateau at 4 mpi in Tg mice inoculated with αsvn PFFs. Scale bar = 200 µm. (B) The high-power magnification of the area enclosed by the square in (A). Note that most of the αsynGFP and pαsyn-positive aggregates did not merge. Scale bar = 100 µm. (C) With increased sensitivity, fluorescence micrographs of GFP and immunostaining with anti-pαsyn (red) antibody in the striatum and corpus callosum of Tg mice inoculated with αsyn-PFFs. The merged images include DAPI (blue). Arrows indicate weak but detectable pαsyn signals of αsynGFP aggregates in the striatum of the Tg mice at 1 and 4 mpi. Scale bar = 20 µm. mpi, month(s) post-inoculation; Ctx, cortex; CC, corpus callosum; Str, striatum; PFFs, preformed fibrils; pαsyn, phosphorylated α-synuclein.

Additional file 4: Figure S4. Immunohistochemical staining of human brains with anti-pαsyn, CNP, MAP2, and NF antibodies. The merged images include DAPI (blue). Lewy neurites in the DLB brain did not co-localize with CNP. No pαsyn-positive aggregates were observed in the control brain. Scale bar = 10 µm. DLB, dementia with Lewy bodies; C, control; NF, neurofilament.

Additional file 5: Table S1. Clinical information for autopsy cases. Table S2. List of primary antibodies used for immunohistochemistry.

## Data Availability

The datasets used and/or analyzed during the current study are available from the corresponding author upon reasonable request.

## Abbreviations

αsyn: Alpha-synuclein
BGH: Bovine growth hormone
BH: Brain homogenate
CD68: Cluster of differentiation 68
CNP: 2’, 3’-Cyclic nucleotide 3’-phosphodiesterase
DLB: Dementia with Lewy bodies
GCIs: Glial cytoplasmic inclusions
GFAP: Glial fibrillary acidic protein
GFP: Green fluorescent protein
GSTpi: Glutathione S transferase pi
Iba1: Ionized calcium-binding adapter molecule 1
IHC: Immunohistochemistry
MAP2: Microtubule-associated protein 2
MSA: Multiple system atrophy
NeuN: Neuronal nuclear antigen
NF: Neurofilament
OLGs: Oligodendrocytes
pαsyn: Phosphorylated alpha-synuclein
PBS: Phosphate-buffered saline
PD: Parkinson's disease
PFFs: Preformed fibrils
PK: Proteinase K
p62: P62/sequestrosome-1
TBS: Tris-buffered saline
TBST: Tris-buffered saline with Tween 20
Tg: Transgenic
TPPP: Tubulin polymerization promoting protein
Ub: Ubiquitin
Wt: Wild type
