# Supplementary figures and images for: A novel mouse model for investigating α-synuclein aggregates in oligodendrocytes: implications for the glial cytoplasmic inclusions in multiple system atrophy
Source: Mol Brain. 2024 May 24;17:28. doi: 10.1186/s13041-024-01104-7 (PMC11127389; doi:10.1186/s13041-024-01104-7)

**A**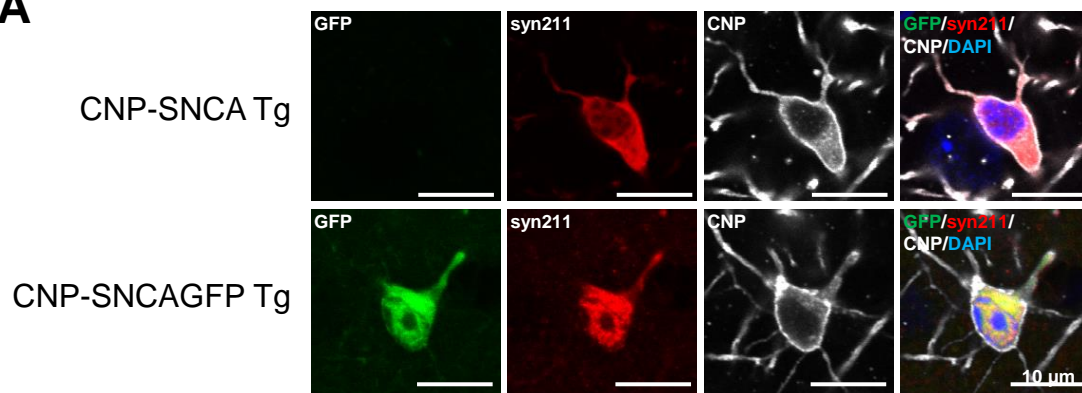**B**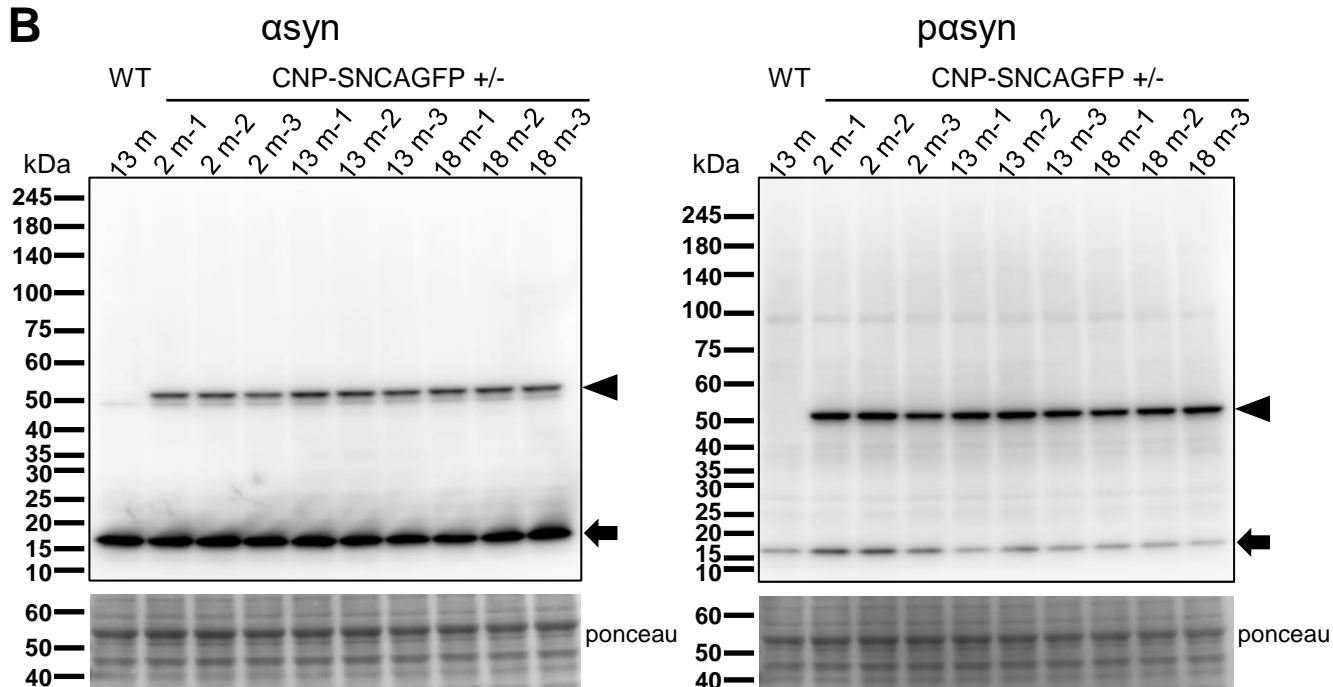**C**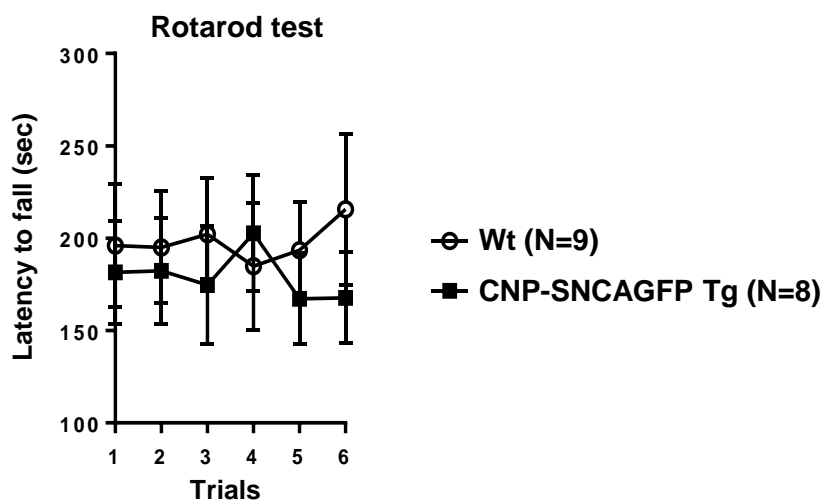

Supplement: Supplementary file 1 — Additional file 1: Fig. S1. The αsynGFP in CNP-SNCAGFP Tg mice exhibits similar subcellular distribution to αsyn in CNP-SNCATg mice and does not aggregate with age. (A) Fluorescence micrographs of GFP and immunostaining with anti-human αsyn antibodies (red) and CNP (gray) of the striatum in CNP-SNCAGFP Tg mice and in CNP-SNCA Tg mice that express human αsyn in OLGs. Scale bar = 10 µm. (B) Western blot images of RIPA-soluble fraction with anti-αsyn and pαsyn antibodies in whole brains of Wt and Tg mice (2, 13, and 18 months old). The expression of endogenous mouse αsyn (arrow) and transgenic human αsynGFP fusion protein (arrowhead) are shown. (C) Latency to fall in the rotarod test at 12 months old. Student’s t-test was performed (not significant). Data indicate the mean ± SEM. Wt, wild-type; αsyn, alpha-synuclein; pαsyn, phosphorylated α-synuclein; PK, proteinase K; RT, room temperature. [file 13041_2024_1104_MOESM1_ESM.pdf]

**A**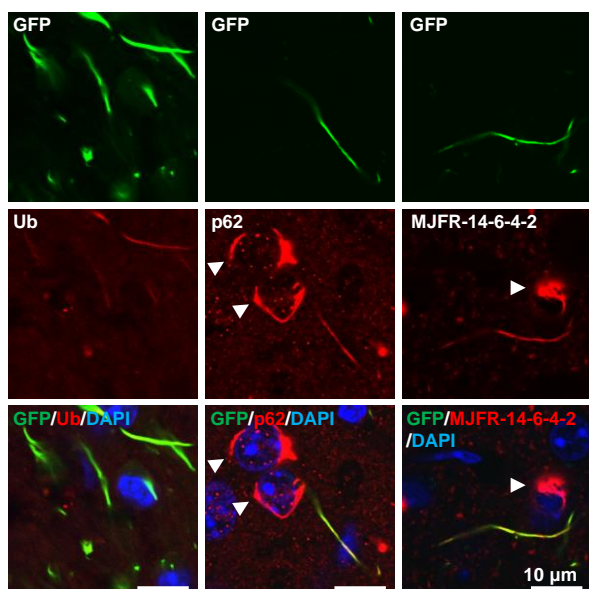**B**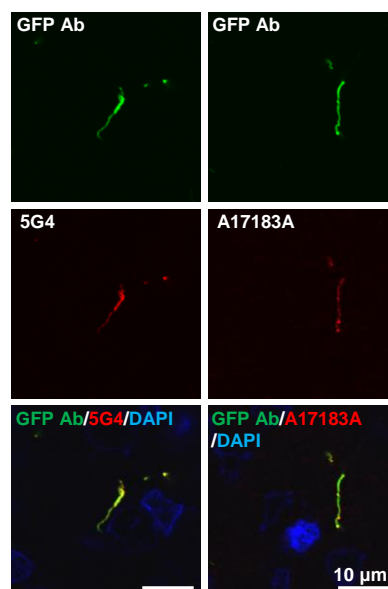**C**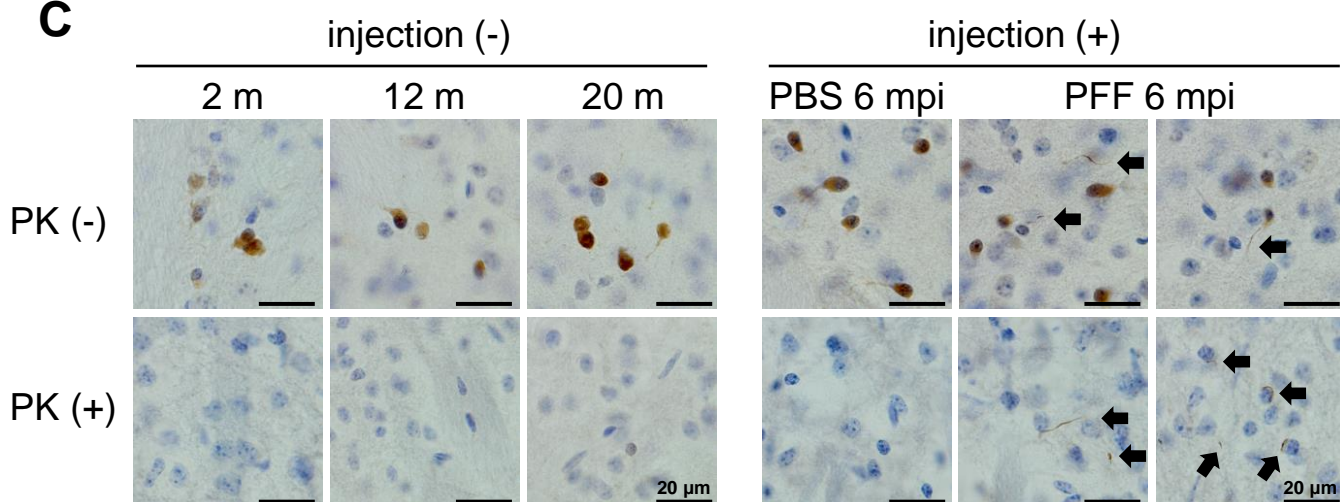

Supplement: Supplementary file 2 — Additional file 2: Fig. S2. GFP dot signals co-localized with markers of αsyn aggregates. (A) Fluorescence micrographs of GFP and immunostaining with anti-ubiquitin, p62, and aggregated form of αsyn antibodies (MJFR-14–6-4–2) in the striatum of Tg mice at 6 mpi of αsyn PFFs. The merged images include DAPI (blue). Aggregates that were immunopositive for p62 and MJFR-14–6-4–2 that did not co-localized with GFP were neuronal (arrowheads). (B) Immunohistochemical staining with anti-GFP and aggregated form of αsyn antibodies (5G4 and A17183A) in the striatum of Tg mice at 6 mpi of αsyn PFFs (paraffin-embedded sample). The merged images include DAPI (blue). Scale bar = 10 µm. (C) Immunohistochemical staining with human-specific αsyn antibody (MJFR1) in the striatum of 2, 12, and 20 months-old Tg mice (left panel) and in the striatum of Tg mice at 6 mpi of PBS or αsyn PFFs (right panel). The lower panel shows sections stained under the same conditions as the upper panel after PK digestion (200 µg/ml, 30 min, RT). Arrows represent αsyn aggregates in OLGs. Scale bar = 20 µm. Ub, ubiquitin; PK, proteinase K; RT, room temperature. [file 13041_2024_1104_MOESM2_ESM.pdf]

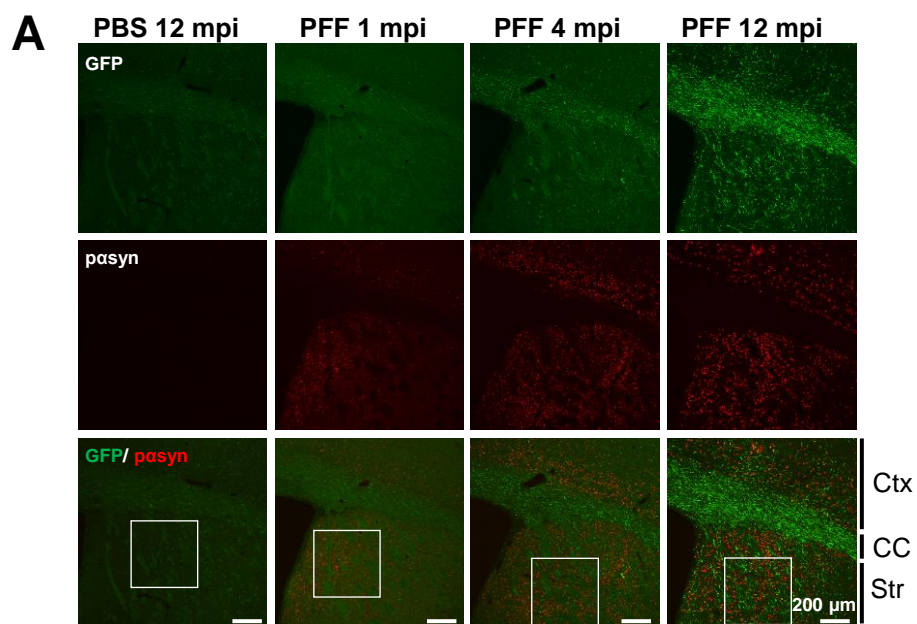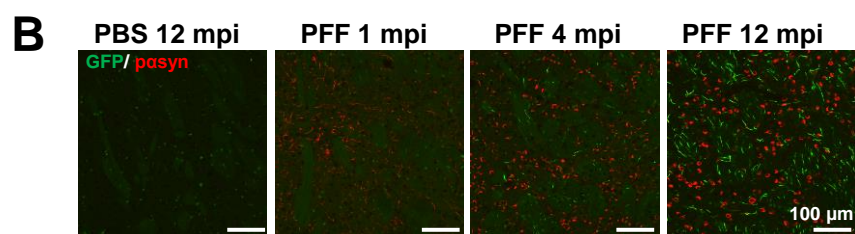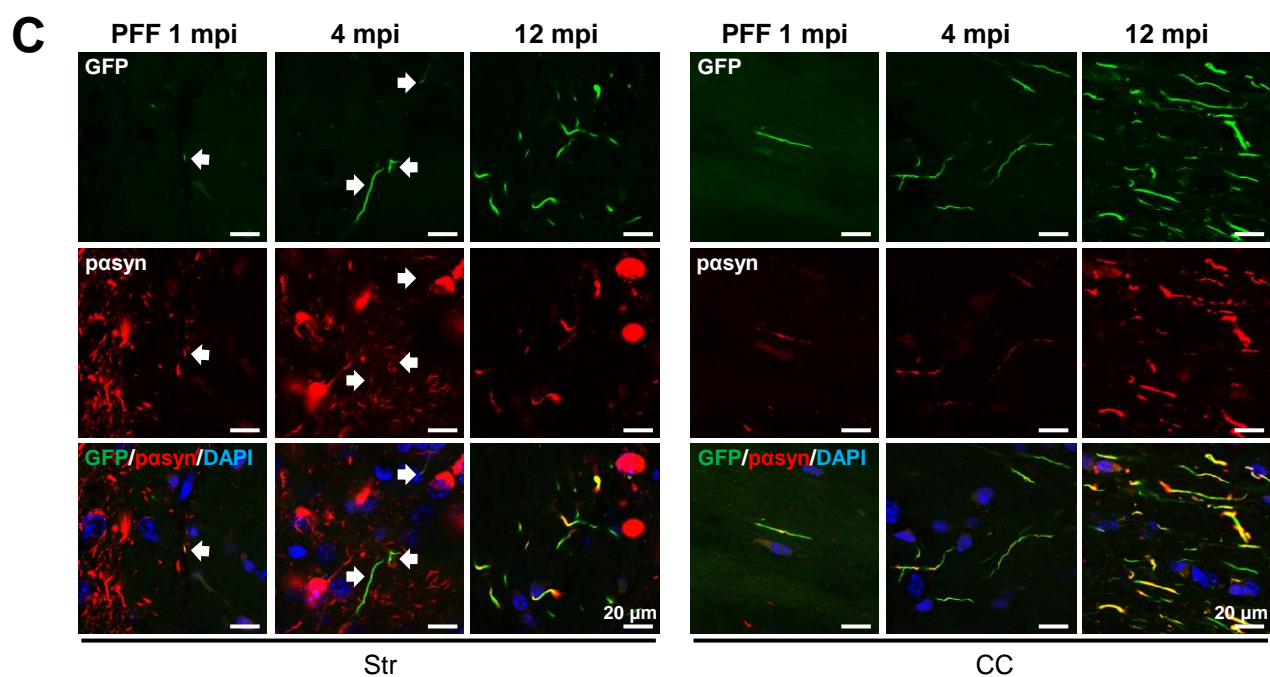

Supplement: Supplementary file 3 — Additional file 3: Fig. S3. Sensitive detection of oligodendroglial aggregates by αsynGFP signals in CNP-SNCAGFP Tg mice treated with αsynPFFs. Fluorescence micrographs of GFP and immunostaining with pαsyn antibodies in Tg mice (low-power magnification). The signal intensity of GFP dots increased over time until 12 mpi, especially in the CC, whereas that of pαsyn reached a plateau at 4 mpi in Tg mice inoculated with αsvn PFFs. Scale bar = 200 µm. (B) The high-power magnification of the area enclosed by the square in (A). Note that most of the αsynGFP and pαsyn-positive aggregates did not merge. Scale bar = 100 µm. (C) With increased sensitivity, fluorescence micrographs of GFP and immunostaining with anti-pαsyn (red) antibody in the striatum and corpus callosum of Tg mice inoculated with αsyn-PFFs. The merged images include DAPI (blue). Arrows indicate weak but detectable pαsyn signals of αsynGFP aggregates in the striatum of the Tg mice at 1 and 4 mpi. Scale bar = 20 µm. mpi, month(s) post-inoculation; Ctx, cortex; CC, corpus callosum; Str, striatum; PFFs, preformed fibrils; pαsyn, phosphorylated α-synuclein. [file 13041_2024_1104_MOESM3_ESM.pdf]

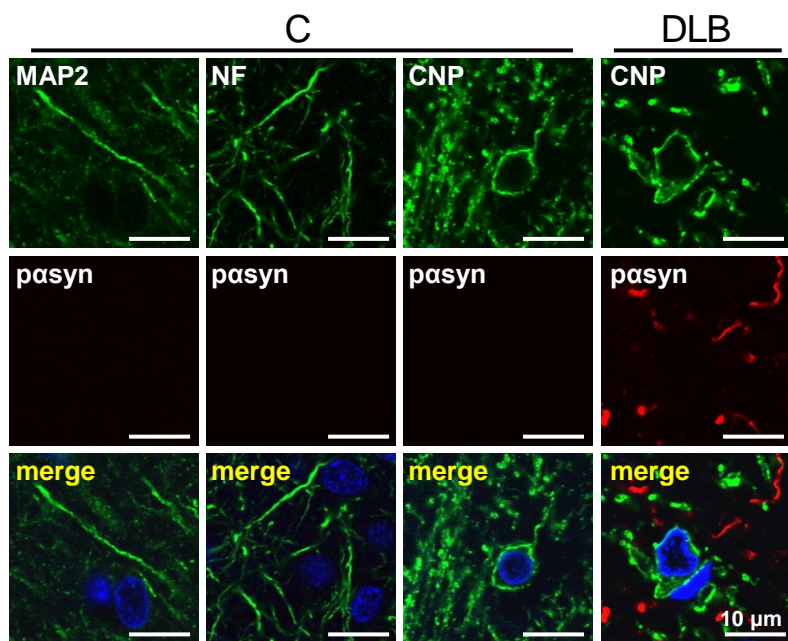

Supplement: Supplementary file 4 — Additional file 4: Figure S4. Immunohistochemical staining of human brains with anti-pαsyn, CNP, MAP2, and NF antibodies. The merged images include DAPI (blue). Lewy neurites in the DLB brain did not co-localize with CNP. No pαsyn-positive aggregates were observed in the control brain. Scale bar = 10 µm. DLB, dementia with Lewy bodies; C, control; NF, neurofilament. [file 13041_2024_1104_MOESM4_ESM.pdf]
