# Supplementary material for: A novel mouse model for investigating α-synuclein aggregates in oligodendrocytes: implications for the glial cytoplasmic inclusions in multiple system atrophy
Source: Mol Brain. 2024 May 24;17:28. doi: 10.1186/s13041-024-01104-7 (PMC11127389; doi:10.1186/s13041-024-01104-7)
Supplement: Supplementary file 5 — Additional file 5: Table S1. Clinical information for autopsy cases. Table S2. List of primary antibodies used for immunohistochemistry. [file 13041_2024_1104_MOESM5_ESM.docx]

**Table S1.** Clinical information for autopsy cases.

| Case | Diagnosis | Age | Sex | Disease duration (yrs) | PMI (hrs) |
| --- | --- | --- | --- | --- | --- |
| M1 | MSA-P | 71 | Male | 2 | 3 |
| M2 | MSA-C | 71 | Female | 4 | 8 |
| M3 | MSA-C | 78 | Female | 3 | 2 |
| M4 | MSA-C | 60 | Male | 5 | 1.5 |
| M5 | MSA-C | 82 | Female | 20 | 2 |
| M6 | MSA-C | 76 | Male | 7 | 6 |
| M7 | MSA | NA | NA | NA | NA |
| D1 | DLB | 81 | Male | NA | 9 |
| D2 | DLB | 89 | Female | 10 | 2 |
| D3 | DLB | 85 | Female | 8 | 9 |
| D4 | DLB | NA | NA | NA | NA |
| C | Cerebral infarction | 86 | Female | NA | 4 |

NA: not available, PMI: post-mortem interval, yrs: years, hrs: hours

**Table S2.** List of primary antibodies used for immunohistochemistry.

| Antibodies | Antigen | Catalog number | Dilution |
| --- | --- | --- | --- |
| Olig2 | Oligodendrocyte transcription factor 2 | Millipore AB9610 | 1:200 |
| CNP | Human CNP | Millipore MAB326 | 1:200 |
| CNP | Human CNP | ATLAS Antibody HPA023280 | 1:500 |
| TPPP | Tubulin polymerization promoting protein | Abcam ab92305 | 1:200 |
| GSTpi | Glutathione S-transferase pi | MBL 312 | 1:200 |
| NeuN | Neuronal nuclear antigen | Millipore ABN78 | 1:500 |
| NeuN | Neuronal nuclear antigen | Millipore ABN91 | 1:200 |
| Neurofilament | Neurofilament | BioLegend SMI312 | 1:200 |
| GFAP | Glial fibrillary acidic protein | Abcam ab53554 | 1:200 |
| GFP | Green Fluorescent Protein | Invitrogen A-11122 | 1:1,000 |
| Iba1 | Ionized calcium-binding adapter molecule 1 | Wako 019-1974 | 1:500 |
| phosphorylated αsyn | αsyn phosphorylated at Ser 129 | Abcam ab51253 | 1:5,000 |
| phosphorylated αsyn | αsyn phosphorylated at Ser 129 | Wako #64 | 1:500 |
| p62 | Sequestosome 1 (p62/SQSTM1) | MBL PM045 | 1:500 |
| Ubiquitin | Ubiquitin | Dako Z0458 | 1:200 |
| LB509 | Human αsyn | Invitrogen 180215 | 1:100 |
| syn211 | Human αsyn | Abcam ab80627 | 1:500 |
| MJFR1 | Human αsyn | Abcam ab209420 | 1:500 |
| MJFR-14-6-4-2 | αsyn aggregate | Abcam ab209538 | 1:5,000 |
| αsyn aggregate | Human αsyn aggregate | Millipore MAB389 | 1:200 |
| αsyn aggregate | Human αsyn aggregate | Biolegend 864902 | 1:200 |
